# A Rare Ectopic Ovary Mimicking Colon Sigmoideum Mesenchymoma Presenting as an Intestinal Mesenchymoma

**DOI:** 10.3389/fonc.2019.00580

**Published:** 2019-07-02

**Authors:** Jiyong Pan, Shuang Wang, Hai Wang, Zhe Fan

**Affiliations:** ^1^Department of General Surgery, The Third People's Hospital of Dalian, Dalian Medical University, Dalian, China; ^2^Department of Endocrinology, The Second Affiliated Hospital of Dalian Medical University, Dalian, China; ^3^Department of Pathology, The Third People's Hospital of Dalian, Dalian Medical University, Dalian, China

**Keywords:** ectopic ovary, colon sigmoideum mesenchymoma, intestinal mesenchymoma, abdominal pain, GISTs

## Abstract

Ectopic ovaries are a rare occurrence. A 33-year-old woman presented to our unit for evaluation of a 2-year history of sporadic abdominal pain that was becoming sharp and frequent. Computed tomography (CT) suggested a gastrointestinal tract mesenchymoma. An abdominal laparotomy was performed and the tumor was excised for pathologic evaluation. A rapid frozen section pathologic examination showed a solitary fibrous tumor (SFT). The final pathology report was an ectopic ovary with corpora lutea bleeding. Ectopic ovaries are benign and the present case is the first report involving an ectopic ovary mimicking a gastrointestinal stromal tumor (GIST). The patient recovered well after surgery. Maldevelopment of the genital tract can lead to ectopic ovaries and surgery is a good management choice. The present case provides a possible differential diagnosis for GISTs.

## Introduction

Ectopic ovaries are rare embryologic abnormalities with an estimated prevalence between 1:29,000 and 1:93,000 gynecologic admissions (1, 2). Because patients are asymptomatic, it is difficult to diagnose ectopic ovaries (3). Gastrointestinal stromal tumors (GISTs) are rare tumors which can arise anywhere within the GI tract (4). Herein, we report the first case of a patient with an ectopic ovary presenting as a GIST and provide the differential diagnosis for GIST.

## Case presentation

**Figure 1:**
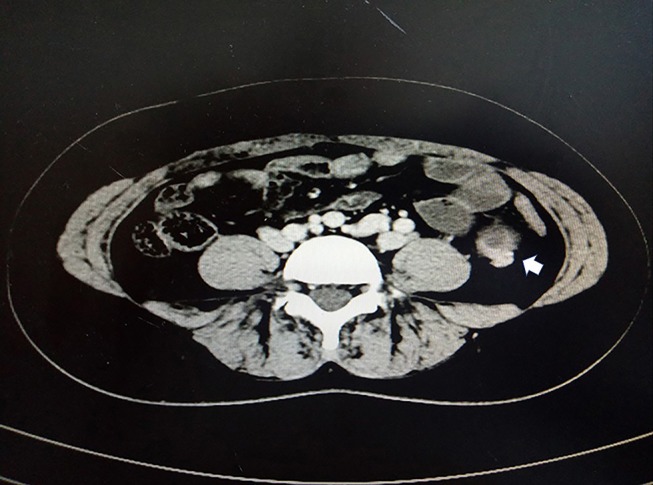
Contrast-enhanced computed tomography (CT) showing a cystic mass in the left lower quadrant (white arrow).

**Figure 2:**
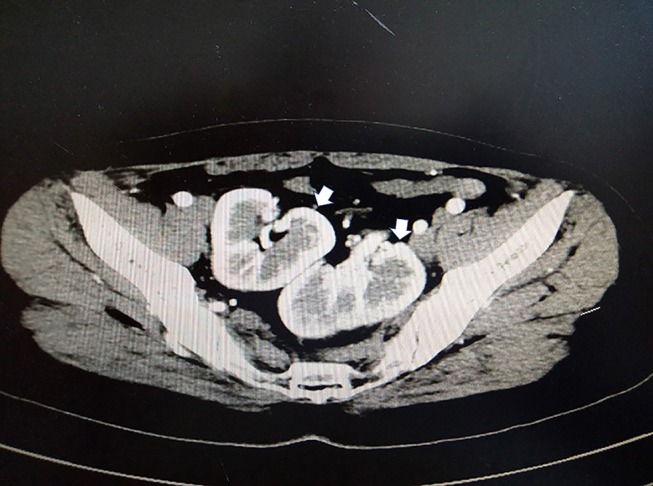
Contrast-enhanced CT presenting bilateral kidneys located in the pelvic cavity (white arrow).

**Figure 3:**
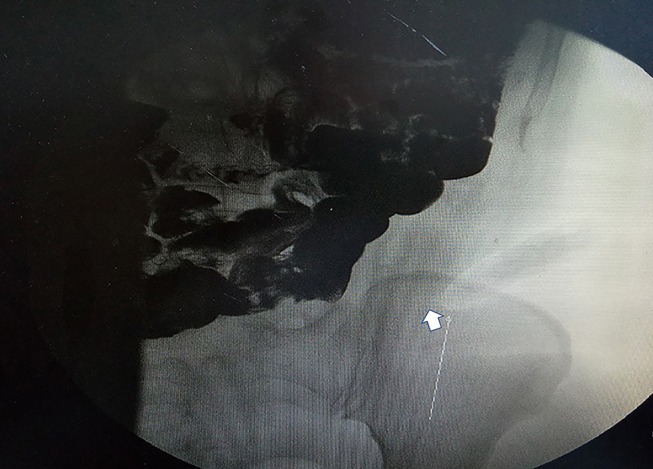
Gastrointestinal radiography revealing a mass in the left lower quadrant. Arrow points to an oval tumor (white arrow).

**Figure 4:**
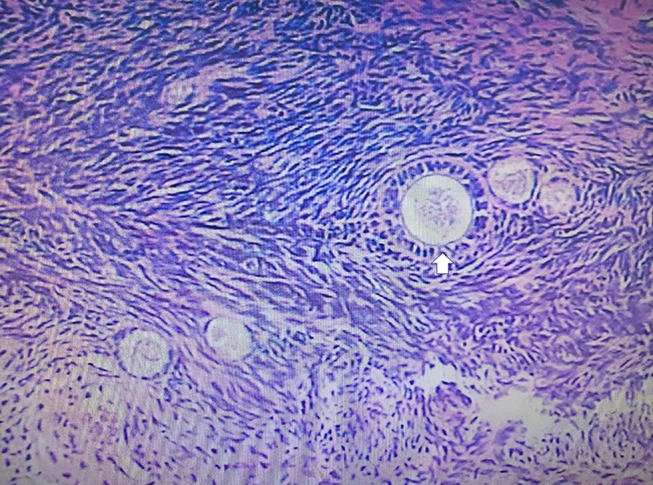
The pathologic examination demonstrated a gray-red cystic mass with blood. The histologic examination revealed an ectopic ovary with corpora lutea bleeding (white arrow).

A 33-year-old female sought evaluation in our Department of General Surgery with a 2-year history of sporadic abdominal pain that had become aggravated during the past week. The character of pain became sharp and frequent. The pain was localized to the left lower abdomen. There was no nausea and vomiting. There was no history of abdominal trauma. The patient had a congenital anomaly of the kidneys and uterus; there was no menstruation. The patient had undergone an appendectomy in the past. On physical examination, the patient was afebrile. The abdominal examination revealed pain and a mass in the left lower quadrant area upon palpation. The mass was approximately 4 × 5 cm in diameters and was not circumscribed. The patient had no rebound tenderness and muscle rigidity. Laboratory testing revealed the following: white blood cell count, 7.13 × 10^9^/L; neutrophilic granulocytes, 76.8%; hemoglobin, 120 g/L; and platelet count, 322 × 10^9^/L. Computed tomography (CT) revealed an intestinal stromal tumor (Figure 1) and pelvic kidneys (Figure 2). Digestive tract radiography showed possible extraintestinal involvement (Figure 3). An intestinal stromal tumor was diagnosed and an abdominal laparotomy was performed; however, the intestinal tract was normal and a mass was noted in the sigmoid flexure. The tumor exhibited exophytic growth without infiltration and was 6.0 × 5.0 × 3.0 cm in size. The tumor and colon (proximal and distal length, 10 cm; ~25 cm) were excised. A rapid frozen section pathologic examination revealed a solitary fibrous tumor (SFT). A colon anastomosis was performed and the patient had fully recovered 7-days post-operatively. The final diagnosis was an ectopic ovary with corpora lutea bleeding (Figure 4). The patient recovered well after surgery and there were no post-operative complications. The patient was doing well at the 11-month follow-up visit. Written informed consent was obtained from the patient and The Third People's Hospital of Dalian had approved the study (NO. 2018-LW-001).

## Discussion

The patient presented to the hospital for evaluation of aggravated abdominal pain, and the CT scan revealed an intestinal stromal tumor. Intra-operatively, a mass located in the colon was thought to be a colon stromal tumor; however, the final pathologic diagnosis was an ectopic ovary with corpora lutea bleeding.

Ectopic ovaries can be classified as congenital and acquired (5). The present case belongs to the congenital type. A developmental error occurring during the formation of genital canals and external genitalia in women may induce ectopic ovaries (6, 7).

The methods by which ectopic ovaries are diagnosed include MRI and surgery; however, surgery is the gold standard (3, 8). MRI can be used to diagnose genital tract and renal system abnormalities (8). Controlled ovarian stimulation (COH) is thought to aid in the diagnosis of ectopic ovaries; magnetic resonance imaging (MRI) more accurately identifies undescended ovaries in the upper abdomen after COH (9, 10). In the present study, because the CT scan revealed an intestinal stromal tumor, an MRI was not performed. Ectopic ovaries are usually accompanied by maldevelopment of the genital system and renal tract (11). The present case had similar maldevelopments: congenital abnormal development of the ovaries and ectopic kidneys. Ectopic ovaries may lead to menstrual disorders, infertility, or abdominal pain (3). In the present case, because of uterine dysfunction and amenorrhea, an ectopic ovary was not suspected. Ectopic ovaries can be found in the upper abdomen, near the pelvic brim or neighboring inguinal canal. The location of the ovary in the current case was the colon, which is the first such reported case.

GISTs are gastrointestinal mesenchymal tumors accounting for 0.2% of all gastrointestinal tumors (12). GISTs can originate anywhere in the gastrointestinal tract. Therefore, the present case was initially suspected to be a GIST.

Ectopic ovaries can present as primary infertility (13), a hernia or cyst in the inguinal canal (14), acute appendicitis (3), ovarian malignancy (15), a Brenner tumor (16), a Wilms' tumor (17), as well as a GIST. Ectopic ovaries can cause irregular menses and pain (18), and are often accompanied by an abnormal urinary system (11) or a mature teratoma (19).

Patients with developmental anomalies need close attention. An abnormal urinary system is usually accompanied by an abnormal genital system. Although it is difficult for the diagnosis of ectopic ovaries pre-operatively, additional examinations should be performed, such as ultrasonography, MRI, and endoscopy. A multiple disciplinary team (MDT) is also advised.

## Conclusion

The present case is an ectopic ovary mimicking a GIST. Maldevelopment of the genital tract can lead to an ectopic ovary and surgery is a good management choice. We have shared our clinical experience to help guide the management of similar cases and offer a differential diagnosis of GISTs.

## Data Availability

All datasets generated for this study are included in the manuscript and/or the supplementary files.

## Consent

Written informed consent was obtained from the patient for publication of this case report and the accompanying images.

## Author Contributions

JP and SW: conceptualization. HW: data curation. ZF: investigation, validation, and writing of the original draft.

### Conflict of Interest Statement

The authors declare that the research was conducted in the absence of any commercial or financial relationships that could be construed as a potential conflict of interest.
